# Occupational and community risk of SARS-CoV-2 infection among employees of a long-term care facility: an observational study

**DOI:** 10.1186/s13756-022-01092-0

**Published:** 2022-03-18

**Authors:** Lauriane Lenggenhager, Romain Martischang, Julien Sauser, Monica Perez, Laure Vieux, Christophe Graf, Samuel Cordey, Florian Laubscher, Tomás Robalo Nunes, Walter Zingg, Anne Cori, Stephan Harbarth, Mohamed Abbas

**Affiliations:** 1 Infection Control Programme, Geneva University Hospitals, 4 Rue Gabrielle Perret-Gentil, 1211 Geneva 14, Switzerland; 2 Faculty of Medicine, University of Geneva, Geneva, Switzerland; 3 Laboratory of Virology, Department of Diagnostics, Geneva University Hospitals, Geneva, Switzerland; 4 MRC Centre for Global Infectious Disease Analysis, Imperial College London, London, UK; 5 Occupational Health Service, Geneva University Hospitals, Geneva, Switzerland; 6 Department of Rehabilitation and Geriatrics, Geneva University Hospitals, Geneva, Switzerland; 7 Infectious Diseases Service of Hospital Garcia de Orta, EPE, Almada, Portugal

**Keywords:** SARS-CoV-2, COVID-19, Nosocomial outbreaks, Occupational exposure

## Abstract

**Background:**

We investigated the contribution of both occupational and community exposure for severe acute respiratory syndrome coronavirus 2 (SARS-CoV-2) infection among employees of a university-affiliated long-term care facility (LTCF), during the 1^st^ pandemic wave in Switzerland (March–June 2020).

**Methods:**

We performed a nested analysis of a seroprevalence study among all volunteering LTCF staff to determine community and nosocomial risk factors for SARS-CoV-2 seropositivity using modified Poison regression. We also combined epidemiological and genetic sequencing data from a coronavirus disease 2019 (COVID-19) outbreak investigation in a LTCF ward to infer transmission dynamics and acquisition routes of SARS-CoV-2, and evaluated strain relatedness using a maximum likelihood phylogenetic tree.

**Results:**

Among 285 LTCF employees, 176 participated in the seroprevalence study, of whom 30 (17%) were seropositive for SARS-CoV-2. Most (141/176, 80%) were healthcare workers (HCWs). Risk factors for seropositivity included exposure to a COVID-19 inpatient (adjusted prevalence ratio [aPR] 2.6; 95% CI 0.9–8.1) and community contact with a COVID-19 case (aPR 1.7; 95% CI 0.8–3.5). Among 18 employees included in the outbreak investigation, the outbreak reconstruction suggests 4 likely importation events by HCWs with secondary transmissions to other HCWs and patients.

**Conclusions:**

These two complementary epidemiologic and molecular approaches suggest a substantial contribution of both occupational and community exposures to COVID-19 risk among HCWs in LTCFs. These data may help to better assess the importance of occupational health hazards and related legal implications during the COVID-19 pandemic.

**Supplementary Information:**

The online version contains supplementary material available at 10.1186/s13756-022-01092-0.

## Introduction

Long-term care facility (LTCF) healthcare workers (HCWs) are recognized vectors in the transmission chain between other HCWs and residents [1–3]. As most studies of transmission routes and risk factors for employee seroconversion were studied in acute-care settings [4, 5], evidence remains scarce for LTCFs, often weakened by flawed surveillance data [6].

In many countries, including Switzerland, COVID-19 among employees working in healthcare institutions caring for COVID-19 patients is automatically recognized as an occupational disease [7]. Nevertheless, the community contribution to SARS-CoV-2 acquisition among health sector employees and in nosocomial outbreaks has recently increasingly been pointed out [1, 4, 7–12]. To our knowledge, the exact contribution of community exposures and occupational health hazards leading to SARS-CoV-2 infection among HCWs in LTCF or nursing home settings has not yet been determined.

Geneva University Hospitals (HUG) affiliated LTCFs are particularly befitted to estimate SARS-CoV-2 acquisition modes and infection rates among employees given the implementation of a robust surveillance system during the first pandemic wave. This includes (1) an institution-wide seroprevalence survey, (2) an outbreak investigation among LTCF patients and HCWs, (3) systematic, syndromic surveillance of employees, and (4) the systematic storage of viral isolates by the National Center of Emerging Viral Diseases hosted at HUG. Here we combine these epidemiologic, molecular, serological and genotypic data collected in the same LTCF to understand the contribution of both occupational and community exposure for COVID-19 infection among employees of a university-affiliated LTCF in Switzerland.

## Methods

### Study design and population

In this cohort study conducted from March 1 to June 30, 2020, in a HUG-affiliated LTCF, we combined data from (1) a previously published institution-wide prospective seroprevalence study in employees, in order to describe the contribution of community and occupational exposure on SARS-CoV-2 seropositivity [13] and (2) an outbreak investigation among HCWs and patients to examine the main transmission dynamic and pathways. The epidemiological situation in Geneva at the time of this study (number of SARS-CoV-2 infections among community and hospitalized COVID-19 cases) can be found in the official public health database (https://www.ge.ch/document/19696/annexe/75).

We included a subgroup of LTCF employees among the 3′241 volunteering participants of the seroprevalence study [13]. Each employee had to attend 3 visits, V1 (baseline), V2 and V3 (see eAppendix 1) from April to June 2020. The outbreak in the selected ward of the LTCF spanned from March 15 to April 8 (based on swab dates) and involved 12 patients (all nosocomial cases) and 23 HCWs, with 10 and 18 specimens analysed by whole genome sequencing (WGS), respectively. HCWs working in the LTCF with a positive SARS-CoV-2 RT-PCR and patients with nosocomial SARS-CoV-2 acquisition were included in the outbreak analysis. All positive samples yielded the wild type SARS-CoV-2 variant, which was the only variant circulating in Geneva during this study.

### Study setting

HUG is the largest tertiary-care centre in Switzerland with > 2000 beds and roughly 13′600 employees. It includes 8 campuses, 4 of which are part of the Department of Rehabilitation and Geriatrics and includes several LTCFs. Our study focuses on a LTCF which includes 8 wards, 4 dedicated to rehabilitation (104 beds) and 4 dedicated to patients awaiting nursing home placement (112 beds), with 285 employees.

### Outcomes and definitions

The primary outcome was SARS-CoV-2 seropositivity among employees, with community and occupational risk factors as primary exposures of interest. As secondary outcome, we investigated transmission pathways (reconstructing the outbreak and determining who infected whom).

Occupational risk factors were classified as any exposure that may result from the performance of an employee’s duties (see Additional file 1: eAppendix 1 and Table 1). Regarding the outbreak reconstruction, patients with nosocomial Covid-19 were included if they had a positive SARS-CoV-2 RT-PCR and onset of symptoms ≥ 5 days after admission in the LTCF, in accordance with Swissnoso guidelines [14]. This study was performed in accordance with the STROBE statement for cohort studies [15] and the ORION guidelines [16].

**Table 1:** Clinical and demographic characteristics of LTCF employees participating in the seroprevalence study

	All participants, no (%)	Seropositive, no (%)	Seronegative, no (%)	*P* value^a^
	176	30 (17)	146 (83)	
Demographics				
Age, mean (SD)	45 (11)	42 (12)	46 (11)	.121
Gender, female n (%)	136/175 (78)	22 (73)	114 (79)	.630
Community exposure				
Transportation	163			.802
Private (include biking)	130	22 (77)	108 (80)	
Public	33	6 (21)	27 (20)	
No. of household member, med (IQR)	3 (1–4)	3 (1–4)	3 (1–4)	
Close contact in the community with a person positive for SARS-CoV-2 within the prior 20 days	29/162 (18)	8 (28)	21 (16)	.179
Occupational exposure				
Healthcare workers	141 (80)	25 (83)	116 (79)	.803
Professional category				
Nurses	48	7 (23)	41 (28)	
Physicians	9	5 (17)	4 (3)	
Nursing assistants	61	11 (37)	50 (34)	
Allied health professional	23	2 (7)	21 (14)	
Office workers	7	2 (7)	5 (3)	
Hospital cleaners	28	3 (10)	25 (17)	
Work rate (%)	169			.300
≤ 80	78 (46)	10 (36)	68 (48)	
> 80	91 (54)	18 (64)	73 (52)	
Close contact with a patient positive for SARS-CoV-2 (< 1 m) within the prior 20 days	131/169 (78)	27 (90)	104 (75)	.091
Close contact with a healthcare worker positive for SARS-CoV-2 (< 1 m) within the prior 20 days	127/166 (77)	23 (79)	104 (76)	.812
Aerosol-generating procedures within the prior 20 days	28/133 (21)	4 (16)	24 (22)	.595
Flat sharing with another healthcare worker	9/161 (6)	0	9 (7)	.365
Eating at the hospital cafeteria	124/170 (73)	21 (72)	103 (73)	1
Carpooling with healthcare workers	26/160 (16)	2 (7)	24 (18)	.257
IPC^b^ measures				
In case of contact with COVID-19–positive patients				
Use of a respirator (FFP2/N95)	14/163 (9)	2 (7)	12 (9)	1
Use of a surgical mask	117/169 (69)	25 (83)	92 (66)	.081
Clinical data				
Presence of symptoms within the prior 20 day	127/167 (76)	27 (93)	100 (73)	.017
Cough	40/164 (24)	14 (48)	26 (19)	
Fever	36/163 (22)	15 (52)	21 (16)	
Headache	85/164 (52)	21 (72)	64 (47)	
Cold	55/164 (34)	11 (38)	44 (33)	
Sore throat	59/164 (36)	10 (34)	49 (36)	
Myalgia	56/163 (34)	15 (52)	41 (31)	
Underwent PCR testing^c^	56/138 (41)	17 (61)	39 (36)	.018
Positive	15/21 (71)	13 (81)	2 (40)	
Requiring hospitalization	1/10 (10)	1 (10)	0	

^a^Fisher’s exact test

^b^Infection prevention and control

^c^Self-reported

### Data sources

We retrieved LTCF employee data from the institution-wide prospective seroprevalence study, for which health-related data were collected at each visit using a self-completed questionnaire [13]. We retrieved data from patients and HCWs included in the outbreak (dates of symptoms onset, date of positive SARS-CoV-2 RT-PCR) from a prospective national surveillance of all COVID-19 patients mandated by the Swiss Federal Office of Public Heath (FOPH) [17] and from the Department of Occupational Health, respectively.

### Microbiological methods

As previously described, participants’ samples collected for the seroprevalence survey were analysed with a 2-tiered diagnostic strategy [13]. All COVID-19 cases included in the outbreak investigation were confirmed by RT-PCR on naso-pharyngeal swab and followed by WGS using an unbiased high-throughput sequencing method (see Additional file 1: eAppendix 2 for details).

### Statistical analysis

We performed descriptive analyses with means (± standard deviations (SD)) or medians (interquartile range (IQR)) and proportions, as appropriate. For comparisons between groups we used Student's *t*-test and Pearson’s chi-square test or Fisher's exact test, for continuous and categorical variables respectively.

For the seroprevalence analysis, modified Poisson regression with robust variance was performed to determine and estimate risk factors for seropositivity. Given the small number of events and to avoid overfitting, we decided to limit the number of covariates in the statistical model. Variable selection using best subset regression with clinical consideration retained close contact (< 1 m) with a COVID-19 inpatient within the previous 20 days and close contact with a laboratory-proven COVID-19 case in the community within the previous 20 days as independent variables(Additional file 1: eAppendix 3). This model was compared to the null model using a likelihood ratio test and retained if it showed statistical significance. We performed a sensitivity analysis of exposure risks among survey participants at baseline only (visit 1), in order to limit the impact of infection control measures in the community (lockdown) on the analysis.

As previously described [18], the outbreak reconstruction was performed combining epidemiological (date of symptom onset and trajectories) and genetic sequencing data. It includes (1) construction of an epidemic curve [19], (2) an ancestry reconstruction (who infected whom) using the outbreaker2 package [20, 21], (3) a maximum posterior transmission tree, and (4) a maximum likelihood phylogenetic tree.

Analyses were performed with Stata v.15 (StataCorp, College Station, Texas, USA) and R version 4.0.4 (2021-02-15) (R Foundation for Statistical Computing, Vienna, Austria). Detailed methods are described in the Additional file 1: eAppendix 3.

## Results

### Seroprevalence analysis

Among 285 employees working in the LTCF from March 1 to June 30, 2020, 199 (70%) participated in the seroprevalence survey and 176 (62%) were included in our analysis. Twenty-three were excluded because of an unknown serological status at the third visit (see Additional file 1: eAppendix 1 and Figs. S1). Thirty (17%) of the 176 participants were seropositive for SARS-CoV-2. The majority were female (136/175, 78%), HCWs with patient contact (141/176, 80%) and presented ≥ 1 symptom compatible with COVID-19 (127/167, 76%). Data regarding symptoms and gender were missing for 9 and 1 employees, respectively. Seropositive participants were more likely to be exposed to a COVID-19 case in the community (8/30, 28%) and to a COVID-19 patient at work (27/30, 90%) than seronegative participants, with 21/146 (16%) and 104/146 (75%) seronegative participants reporting a contact with a COVID-19 case in the community and with a COVID-19 patient at work, respectively. Clinical and demographic characteristics of employees are described in Table 1.

Multivariable analysis revealed that both occupational exposure (adjusted prevalence ratio aPR 2.6; 95% CI 0.9–8.1) and community exposure (aPR 1.7; 95% CI 0.8–3.5) were associated with higher risk of seropositivity, although these associations were not statistically significant.

When restricting the analysis to participants at baseline only (with the first visit between April 8 and 16, 2020), including 20 (10%) seropositive employees and 179 (90%) seronegative employees, multivariable analysis revealed statistically significant associations between seropositivity and both occupational exposure (aPR 3.6; 95% CI 1.1–11.5, *P* = 0.03) and community exposure (aPR 3.28; 95% CI 1.4–7.8, *P* = 0.007).

### Outbreak investigation

**Fig. 1:**
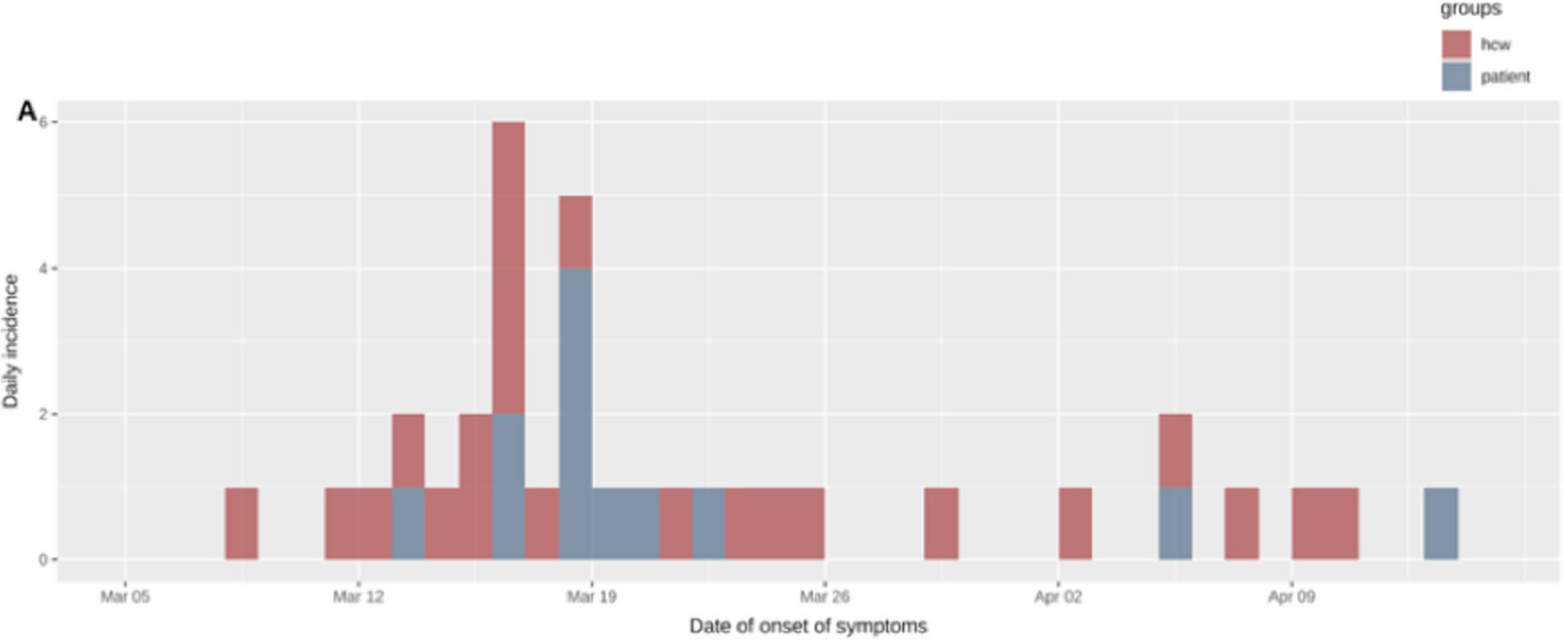
Epidemic curve of the nosocomial COVID-19 outbreak involving LTCF HCWs (red) and patients (blue)

The outbreak spanned from March 15 to April 8, 2020 based on swab dates, with 35 RT-PCR proven cases (12 patients and 23 HCWs). The attack rate was 50% and 23.5% for HCWs and patients, respectively. Figure 1 shows the epidemic curve based on dates of symptom onset.

**Fig. 2:**
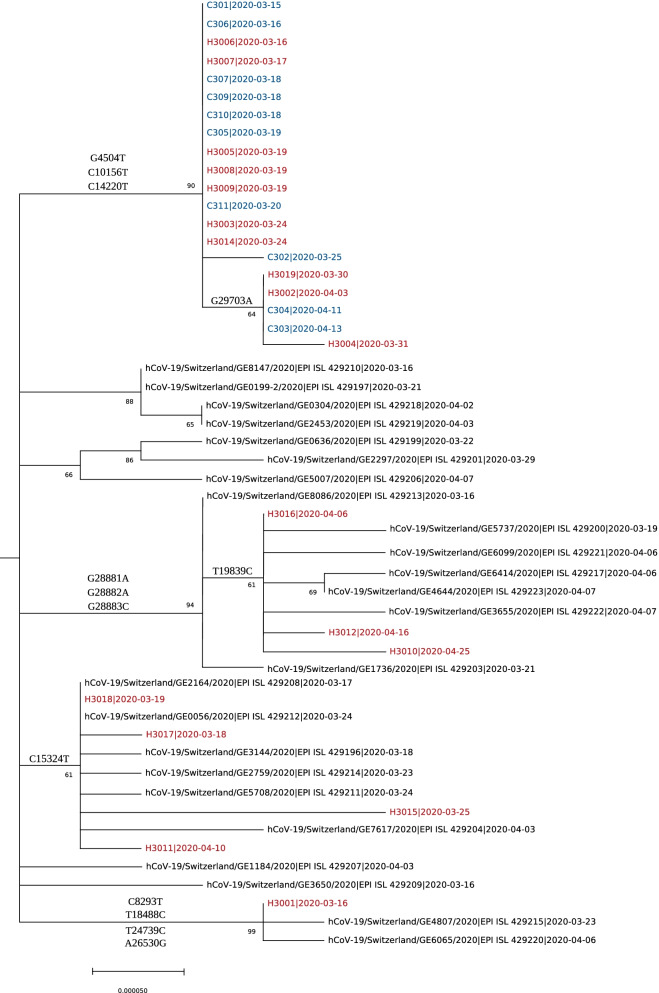
Maximum likelihood phylogenetic tree of SARS-CoV-2 genomes from 10 patients (blue) and 18 HCWs (red) sequenced during the outbreak investigation

Genetic sequences were obtained and a phylogenetic tree was constructed for 18 HCWs (red) and 10 patients (blue) involved in the outbreak (Fig. 2). We observed one large cluster of 10 HCWs and 10 patients (branch names G4504T, C10156T, and C14220T on Fig. 2) with a sub-cluster including 3 HCWs and 2 patients (branch name G29703A on Fig. 2), highly suggestive of nosocomial transmission. Given the sequences and their mutation similarities, it is reasonable to suggest that these 10 HCWs (56%), including one imported case, may have acquired SARS-CoV-2 in the course of their professional activities (clinical and non-clinical duties, commuting together, etc.). The 8 (44%) remaining strains retrieved from HCWs showed a significant amount of genetic diversity, and tend to cluster with community sequences.

Of these 8 strains sharing similarity with community sequences, the ancestry reconstruction highlighted that 4 imported cases among HCWs (H3015, H3017, H3001 and H3016; Additional file 1: Fig. S2) may have secondarily infected 3 other HCWs (H3010, H3012 and H3018), among which one (H3018) secondarily infected another HCWs (H3011), with an estimated posterior support of 100% (H3010, H3012 and H3011) and 58% (H3018) for these transmission links as represented in the maximum posterior transmission tree (Supplementary Fig. 3). Thus, it seems that 4 of the 8 remaining strains from HCWs were infected by other HCWs carrying community sequences.

The numbers of secondary infections per type of case (i.e. HCW versus patient) are shown in Additional file 1: eAppendix 4 and Fig. S4.

## Discussion

This comprehensive study of SARS-CoV-2 acquisition and transmission dynamics conducted in a Swiss LTCF during the 1^st^ pandemic wave shows that both community and occupational exposures play an important role leading to COVID-19 among employees. Overall, these complementary approaches challenge traditional assumptions that SARS-CoV-2 infection in hospital employees is always due to an occupational exposure. Moreover, our results show that the probable 4 imported cases in the initial outbreak were all HCWs harbouring a community sequence (H3015, H3017, H3001, H3016 on Fig. 2, Additional file 1: Fig. S2 and S5), who then secondarily infected their colleagues. Even if the outbreak analysis is unable to confirm that these transmissions between HCWs occurred at work or outside work, our study highlights the important contribution of HCWs and community importation in the transmission chain of this LTCF outbreak of COVID-19.

Our results underline the major role of community-acquired infection in LTCF HCWs, who may then introduce the virus into the facility, and are in line with most studies investigating the occupational health hazard of COVID-19 among hospital employees. Indeed, even though the latter mostly focused on acute care settings, their results also reported a community contribution to SARS-CoV-2 seropositivity among HCWs, with previous household contact with a COVID-19 case as one of the main risk factors [8, 10, 11, 13, 22–26].

Regarding occupational exposure, HCWs, and particularly LTCF HCWs, are known to be at higher risk for SARS-CoV-2 infection than the general population [27–31]. Although this consideration has led to huge efforts to protect HCWs and patients from nosocomial infection [7, 29, 32], this may have led to an overestimation of the importance of patient-to-HCW transmission compared to HCW-to-HCW or community-to-HCW transmission [2, 33].

Our results strongly emphasize the importance of HCW-to-HCWs SARS-CoV-2 transmission, as previously described [18, 34] and thus non only challenge the misperception that patients, are the dominant reservoirs and vectors, but also support the importance of non-pharmaceutical interventions and repeated testing of employees to limit transmission from community to healthcare settings, as well as the importance of aggressive vaccination campaigns among LTCF employees [35–37]. Therefore, occupational exposure is not always related to direct patient care, especially in LTCF where patients have long hospital stays and less risk of importing SARS-CoV-2 from the community.

Disentangling the real occupational and community health hazard of COVID-19 infection among LTCF employees is crucial given the proportion of long COVID syndrome reported in recent studies and the related legal and economic implications. Indeed, in the Swiss legal landscape, the sick leaves due to COVID-19 among employees in contact with patients would be covered by the Federal Law on Accident Insurance and Disability Insurance depending on the duration of the leave, in contrast to Health Insurance when the leave is due to non-occupational disease. Up to two-thirds of infected middle-aged individuals (40–60 years old) may indeed suffer from disabling symptoms potentially lasting for months and leading to long periods of sick leave for 11% of them [38–40].

The main strength of our study is that, to our knowledge, it is the first quantitative analysis of both occupational and community risk exposures of COVID-19 among LTCF employees combining epidemiological, serological, and molecular data. Thus, these findings add precious information regarding SARS-CoV-2 spread in LTCFs, which share many similarities with nursing homes. This study also presents some limitations, including (1) potential selection bias regarding the fact that not all LTCF employees participated in the seroprevalence survey; (2) information and potential recall bias given the retrospective data collection; (3) the fact that contact with an asymptomatic COVID-19 case (community or hospital) may have not been recorded; (4) different populations in the two sets of analysis; (5) lack of power given the small sample size and number of events observed. Finally, despite a non-statistically significant result, and the possibility of residual confounding, the 95% CI of the influence of community on LTCF employees seropositivity clearly indicates a probable exposure effect, which is supported by seroprevalence survey at baseline and detailed genomic analysis.

## Conclusions

In conclusion, these two complementary approaches demonstrate a substantial contribution of both occupational and community exposures to seropositivity and infection risk. The role of HCWs in preventing importation of SARS-CoV-2 to LTCFs from the community is crucial. These data may not only allow to better assess occupational health hazards and related legal implications during and after the COVID-19 pandemic, but also emphasize the urgent need to maximise vaccine uptake in LTCF HCWs in order to limit HCW-to-HCW and HCW-to-patient transmission.

## Supplementary Information

**Additional file 1:** All supplementary data (methods, figures, tables).

## Data Availability

Due to small size of the cluster, clinical data will not be shared in order to safeguard anonymity of patients and HCWs.
